# An Effort Worth Making: A Qualitative Study of How Swedes Respond to Antibiotic Resistance

**DOI:** 10.1093/phe/phaa033

**Published:** 2020-12-03

**Authors:** Mirko Ancillotti, Stefan Eriksson, Tove Godskesen, Dan I Andersson, Jessica Nihlén Fahlquist

**Affiliations:** 1 Uppsala University; 2 Ersta Sköndal Bräcke University College and Uppsala University

## Abstract

Due to the alarming rise of antibiotic resistance, medically unwarranted use of antibiotics has assumed new moral significance. In this paper, a thematic content analysis of focus group discussions was conducted to explore lay people’s views on the moral challenges posed by antibiotic resistance. The most important finding is that lay people are morally sensitive to the problems entailed by antibiotic resistance. Participants saw the decreasing availability of effective antibiotics as a problem of justice. This involves individual as well as collective moral responsibility. Yet, holding agents responsible for their use of antibiotics involves varying degrees of demandingness. In our discussion, these findings are related to the contemporary ethical debate on antibiotic resistance and two proposals for the preservation of antibiotic effectiveness are compared to and evaluated against participants’ views.

## Introduction

Antibiotic resistance (AR) develops as a natural process resulting from the exposure of bacteria to antibacterial drugs and selection for resistant mutants. The rapid development of AR is one of the most significant threats to public health globally (WHO, 2015), which is directly related to how antibiotics are used in society (Costelloe *et al.*, 2010). As the present use of antibiotics drives the evolution of resistance, it also decreases the future availability of effective antibiotics. Thus, the challenge is to keep antibiotic use as low as possible, without compromising therapeutic need, to maximize the therapeutic life span of existing and future antibiotics (Heyman *et al.*, 2014).

The management of AR is a complex public health issue in which many stakeholders play a role. The public can contribute to AR management by not actively seeking the prescription of antibiotics unless recommended by a physician, by adhering to prescriptions and by adopting simple hygiene routines such as hand-washing to reduce the spread of infectious diseases (Lee *et al.*, 2013). Most research has focused on public awareness of the use of antibiotics and AR under the assumption that a better understanding of antibiotics can make people act more responsibly (Brookes-Howell *et al.*, 2012). However, an increase in public knowledge and awareness may be insufficient to effect behavior and attitudinal modifications (Stalsby Lundborg and Tamhankar, 2014; Haenssgen *et al.*, 2018; Carlsson *et al.*, 2019). The problem with a focus on awareness-raising as a behavioral tool is that public campaigns are seldom developed from an adequate appraisal of the attitudes and beliefs that inform social norms that influence antibiotic use (Hawkings *et al.*, 2007; McDonnell Norms Group, 2008; McCullough *et al.*, 2016). Behavioral studies highlight the role of such social norms, which are crucial in bringing about desirable behavior changes (Pinder *et al.*, 2015; Nyborg *et al.*, 2016; Saran *et al.*, 2018). Effective approaches to stewardship should include appropriately targeted awareness campaigns that can positively influence socially conscious citizens (O’Neill *et al.*, 2016).

Some authors use language from economics to describe AR, for example in terms of ‘common goods’. Common goods are non-excludable, in that individuals cannot be excluded from their use, and rivalrous, in that the individual’s use precludes use by others. Rivalry is the consequence of the progressive loss of antibiotic effectiveness and many scholars describe the erosion of antibiotic effectiveness as analogous to the ‘tragedy of the commons’ (Levin, 2001; Baquero and Campos, 2003; Foster and Grundmann, 2006; Heyman *et al.*, 2014; Hollis and Maybarduk, 2015). This concept describes how the exploitation and gradual depletion of a common resource result in a loss of utility distributed equally in the population, while the gain becomes concentrated to the people doing the exploiting. This process was first conceptualized by Garrett Hardin, who illustrated it by farmers overgrazing a shared field to maximize their own benefit at the expense of other farmers (Hardin, 1968). Giubilini (2019) suggested two ethically relevant differences between Hardin’s example and the case of AR. First, antibiotics consumers can become carriers of resistant bacteria, which may lead to the inefficacy of future antibiotic treatment. They will therefore bear the cost of their actions individually. Second, using antibiotics not only contributes to the erosion of a common good but can immediately impact others negatively, as carriers of resistant bacteria can infect others. Therefore, while AR can often be compared to other global challenges such as climate change or biodiversity loss in that individuals in all these cases may lack sufficient motivation to act alone for the sake of the collective, AR has ethically relevant peculiarities that should be acknowledged.

We advocate the idea that social norms hold the potential to contribute to the adoption of judicious antibiotic use through the promotion of virtuous antibiotic use.^1^ Consequently, morally relevant characteristics of the AR problem need to be made explicit. The aim of this study is to explore Swedish lay people’s views on the moral challenges posed by AR. The findings serve as starting point for a discussion on the importance of the various moral characteristics identified and on possible policy proposals for the preservation of antibiotic effectiveness.

A rationale for exploring Swedes’ views on the moral challenges posed by AR is that this population is comparatively more knowledgeable about AR (European Commission, 2016).

## Methods

This study is a secondary analysis of data originally collected with the aim to identify factors promoting and hindering a judicious approach to antibiotics (Ancillotti *et al.*, 2018). Due to the richness of the material and considering that the participants discussed at length the ethical dimension of antibiotic use and AR, another analysis was performed to pursue interests distinct from those of the original analysis (Hinds *et al.*, 1997).

### Design and Settings

An explorative study using focus group discussions (FGDs) was conducted. The interview guide was pilot-tested and structured according to best practice guidelines (Krueger and Casey, 2015). Follow-up and probing questions were used for clarification and elaboration.

The structure (A–E) and themes (within parentheses) of the interviews were: (A) Opening question (introducing oneself and reasons for participating); (B) Introductory question (spontaneous thoughts about antibiotics); (C) Transition question (personal experience of antibiotics); (D) Key questions (advantages and disadvantages of using antibiotics, prescriptions, present and future consequences of the non-judicious use of antibiotics at the individual and community levels, AR, individual responsibility, commitment and cues for action); (E) Ending question (imagining advising health authorities). Participants from the general population were recruited from the city of Uppsala and surrounding areas through a site-based approach and purposive sampling (Arcury and Quandt, 1999). Inclusion criteria were age 18 years or older and proficiency in Swedish. The exclusion criterion was having had medical or health-related education.

All participants received written information before their inclusion and then oral information at the start of the FGDs. The Regional Ethical Review Board in Uppsala, Sweden, approved the study (Dnr 2016/154). To protect participants’ confidentiality, no identifying details are included in this presentation.

### Data Collection

Twenty-three respondents (13 women and 10 men, age range: 20–81) were distributed heterogeneously into four groups according to gender, age and education level (see Table 1). The FGDs took place at Uppsala University in the period October–November 2016. The FGDs lasted between 90 and 120 minutes and a short video containing basic information on AR was shown after about 30 minutes (Nyhetsmorgon, 2016). Participants received a gift card to the value of approximately EUR 25 after participating. No dropouts occurred. The interviews were audio-recorded and transcribed verbatim by a professional transcription service.

**Table 1.** Participants’ demographics

	G 1	G 2		G 3	G 4	
Woman	4	4	3	2	13
Man	3	2	3	2	10
Age					
Minimum				20	
Mean		38			
Maximum		81			
Education^a^					
EQF 4, 5				12	
EQF 6, 7		8			
EQF 8		3			

a European Qualifications Framework (EQF) level: EQF 4, 5 high school, vocational school and university diplomas; EQF 6, 7 Bachelor's degree, vocational universities and Master’s degree; EQF 8 Doctoral degree.

### Data Analysis

The transcripts were coded in QSR International’s NVivo11 Software and analyzed inductively using thematic content analysis (Burnard *et al.*, 2008). In the first stage, two members of the research team independently coded the transcripts. M.A. analyzed all the transcripts and J.N.F analyzed a representative one-fourth of all the group transcripts. To become familiar with the content, the coders read the transcripts multiple times while starting the open coding: the process of identifying themes and categories emerging from the text and taking note of words and phrases that could sum up relevant content.

The second stage meant eliminating duplications and overlapping or too similar categories. In the final stage, sorting the remaining categories into groups refined the distinctions. The analysts then compared their outcomes and critically discussed any inconsistencies. Then the rest of the research team discussed the results to find consensus. The categories are descriptive and render the participants’ terms. The themes are the result of a final abstraction process and are thus interpretative.

## Results

The results describe the moral response of Swedish lay people to the challenges posed by AR as expressed in the context of FGDs. Three main themes ensued from the analysis: justice, responsibility and demandingness (see Table 2). In summary, the participants found that the decreasing availability of effective antibiotics leads to justice-related issues; that the situation implies individual as well as collective moral responsibility and that holding agents responsible involves varying degrees of demandingness.

**Table 2.** Overview of themes and categories and their relative exemplar quotes

Theme	Category	Quote
Justice	Limited resource	Q1
	Questioning the need	Q2
	Distribution criteria	Q3
	Society first	Q4
	Protect everyone’s life	Q5
Responsibility	Moral sentiment	Q6, 7
	Stigma risk	Q8
	Collective responsibility	Q9
	Future generations	Q10
	Individual responsibility	Q11, 12
	Uncertainty of risk	Q13
Demandingness	Increase control	Q14
	Personal struggle	Q15
	Worth the effort	Q16
	Effort not worth making	Q17

### Justice

Considering that the use of antibiotics contributes to AR and that AR decreases the effectiveness of antibiotics, non-judicious use of antibiotics poses a series of justice-related ethical issues:

(Q1) Well, we have a responsibility because it’s kind of unfair that we use up all the resources there are and then there is nothing left… (G3W3)^2^

The participants considered non-judicious use of antibiotics to be unfair and immoral, at both the individual and collective level. They questioned whether patients who use antibiotics always really need them:

(Q2) I probably think like this when I hear the word ‘antibiotics’…when did I last take them… could one have done things differently or… you generally just say yes to them, almost preventively sometimes. (G1W2)

A common concern was where the line should be drawn between justified and unjustified use and who should set the criteria:

(Q3) Who decides whom should be given antibiotics or not? (G1M1)

The participants discussed whether society and societal needs should take precedence over individual interests. The common tendency was to grant priority to societal needs. However, the participants also voiced the concern that prioritizing the collective may bring about the undesired and perhaps fatal consequence of antibiotic treatments being withheld from some patients:

(Q4) Yes, if we look at the big picture and think about how serious it’s starting to get, well it is of course that you must…it’s a sacrifice you have to make, I think, to get a better situation. (G4M2)

Society first. (G4W2)

Yes, exactly. (G4M2)

(Q5) And then it feels like, do you want to take that risk of not prescribing antibiotics and then maybe a patient dies. I mean, you might not always be able to know that. (G1W4)

### Responsibility

Participants believed that they, like everyone, share the responsibility for the current AR situation and must contribute to efforts to curb AR. They thought it was a moral responsibility, which can trigger moral sentiments. Abstaining from using antibiotics would result in a kind of relief while non-judicious use would entail a sort of antibiotic shame:

(Q6) [P]eople have to understand that they’re using them correctly when they really need to use them and that they don’t when it’s not necessary so that they don’t get like some kind of anxiety or negative kind of feeling when it’s right, I mean. (G2M1)

(Q7) So then and there one might think… I must have them, I feel so bad. But then afterwards you would definitely be, probably glad about it …I mean, I didn’t die, I feel good now, isn’t it lucky that I didn’t contribute to it? (G1M1)

Nonetheless, the participants were aware of the risk of stigmatizing people who use antibiotics:

(Q8) It can’t be that you’re stigmatized for taking antibiotics either, because they are going to be needed. It’s just like you said. You can’t make it into a bad thing to treat what needs to be treated because that’s what happens with a moral panic…black and white. (G2W3)

The participants envisioned both collective and individual agency. They argued that there is a responsibility calling for collective action to ensure the effectiveness of antibiotics for those who need them the most, now and in the future. Furthermore, they thought that there is a responsibility toward future generations:

(Q9) I think it’s a huge responsibility for the community to somehow… with information but also by creating some kind of rules system because it is too serious a problem to like not do anything at all about it higher up, like at a higher level. (G3M2)

(Q10) It feels like it should be in our interest that future generations will survive. Short-term thinking, in fact. It’s about the environmental part too but it feels very much as if people think about the here and now, but in a hundred years when people will die from trivial things again. People don’t think that far ahead. (G2W3)

The participants also emphasized the potential role of bottom-up initiatives, such as that of citizens as consumers. However, individual responsibility was the primary focus of the discussions. They criticized patients requesting antibiotics too eagerly and doctors who are too ready to prescribe antibiotics. When patients are not convinced, they should ask whether an antibiotic prescription is necessary. People should also use some precautions to minimize the need for antibiotics: they should inform themselves, take measures to prevent exposure to bacteria (e.g. hygiene) and avoid spreading infections when they are sick. Beyond the medical sphere, people are to assume responsibility also for other behaviors that exacerbate the AR problem, such as traveling to countries known to have high AR records, and food consumption (mainly meat):

(Q11) You have a responsibility to like get informed too, find out the facts, I mean, so that you really understand how it works. So that you don’t just kind of go yes, yes, someone has said that then it might be like that. (G2W4)

(Q12) [Y]ou still like have a physical responsibility for your own body. If you don’t take care of it you will catch things and like, it doesn’t have to be morally wrong, but still. I mean, a purely causal responsibility is what I am thinking. (G3M1)

Understanding the future consequences of AR connects with the notion of uncertainty. While being in spatial and temporal proximity to the effects of AR helps people to feel immediate moral responsibility and makes them ready to take action, the uncertainty of the risk posed by AR tends to dilute their moral feelings. For the individual, the consequences of the present non-judicious use of antibiotics and the adoption of behaviors that can negatively influence AR can be too distant. The participants often used the climate change analogy to describe the intangibility of AR:

(Q13) Yes, well, I think that it could be that it’s hard to conceptualize what is going to happen. Like you said, that just now it feels very abstract. I mean, what will happen is so far away that it just sort of ends up as, yes, you shouldn’t take so many antibiotics because you shouldn’t have so many antibiotics, but not because people are going to die from trivial things. You distinguish between what you do now and what’s going to happen later. (G2W1)

### Demandingness

The participants welcomed the possibility of imposing stricter regulations to curb AR. The focus was mostly on medical prescriptions and the livestock industry. Restricting freedom of movement was acceptable as a self-imposed sacrifice but debatable when thought of as a top-down initiative in relation to the collective:

(Q14) I can’t list them all, but this is one of the reasons that you might not think about a lot, when you’re talking about antibiotic resistance. Then I think that, absolutely, society should step in control and regulate. (G4W1)

More austere regulations and a broad conception of responsibility put demands on individuals that may be difficult to cope with:

(Q15) But what if it’s like your big day, that it’s your wedding or your sister’s wedding or something and then you are supposed to sit there with an earache and you can’t, or someone’s funeral and you can’t focus… and what about the sports stars. Are they exempted or…because then they will get sick and can’t train. (G4W1)

Notwithstanding such notions of a struggle, for the most part the attitude was that of a readiness to make personal efforts, i.e. to use antibiotics judiciously and to engage in AR-related judicious behavior. This held true even if those efforts were associated with personal costs, such as foregoing antibiotics and staying home from work longer, revising food and travel habits and even to suffer more pain from non-treatment.

(Q16) But if you have such a responsibility, I mean it includes some kind of sacrifice…For example, Thailand is a very popular destination now at Christmas. But Thailand is one of the main sources of antibiotic resistance in the world; you should not really go there if you take this somewhat seriously. (G4M1)

(Q17) I feel that I want to know that what I do is not in vain… that I renounce to this [antibiotics] now, that I make this painful sacrifice, and this helps the whole community. (G1M1)

The price that participants were not ready to pay to forego antibiotics was putting their own or others’ lives at risk:

(Q18) It’s very important to know that it’s not dangerous and, like, fatal, that’s very important. (G1W1)

## Discussion

In the following, the results are first considered in light of the academic ethical debate on AR and then in light of two proposals for the preservation of antibiotic effectiveness.

### Situating Lay People’s Moral Views

Participants readily beheld the erosion of antibiotic effectiveness as an ethical issue. As the resource is scarce, it would be unfair to consume it and leave limited to no antibiotic treatment options available to those who need them (be they present or future people). In a nutshell, participants placed societal needs before the individual. Findings from a recent US study support the idea that the public can prioritize society over the individual and that such support increases when people understand the social costs of AR (Dao *et al.*, 2019). Thus, it appears as though people account not only for individual risks and benefits but also collective ones.

Participants in this study prioritized social demands for the preservation of antibiotic effectiveness, but were also concerned about individuals’ needs. Tremmel’s description of justice can help interpret this potential inconsistency (Tremmel, 2009 ). Tremmel described three ways of comprehending justice: justice as impartiality, justice as equal treatment of equal cases and unequal treatment of unequal cases, and justice as reciprocity. In this study, the participants’ views seem to be ascribable to the second conceptualization: Patients who have special needs should receive special treatments.

In the ethics literature, the ‘rescue rule’ is often used to interpret physicians’ preferences for antibiotic therapies believed to benefit present patients, rather than future ones (Garau, 2006; Leibovici *et al.*, 2012; Krockow and Tarrant, 2019). While there are also other possible explanations for this preferred line of action, e.g. legal demands upon care services, there is in general a strong moral impetus for helping a person in need here and now and to disregard the abstract group of people possibly affected in the future (McKie and Richardson, 2003). Patients are generally believed to ‘value therapies that will not only cure them, but are easy to take, will resolve their symptoms quickly and enable them to return to their normal activities as soon as possible’ (Wagstaff, 2006: 13). Although tensions between individual and collective needs emerged in the FGDs, participants were not as egotistical and blind to the competing priorities of healthcare as the public is sometimes believed to be. They were positive toward individuals (including themselves) foregoing antibiotics when possible. The participants seemed willing to weigh collective risks and benefits against the needs of vulnerable patients, i.e. patients who really need antibiotics. This finding is consistent with the results of previous surveys of the general population in Sweden, in which the majority of respondents expressed their willingness to abstain from using antibiotics for the common good (Sveriges Kommuner och Landsting, 2015; Carlsson *et al.*, 2019). This study adds the realization that the common good should not be sought at the expense of individual patients who really need treatment. The respondents implicitly adhered to a lay person’s version of the rescue rule. One interesting question is whether the rescue rule, interpreted as our compassionate duties toward people that we know we can rescue, can have a place in approaches to AR management that aim to prioritize society based on considerations of justice. Considering intergenerational justice, this does not necessarily entail intergenerational equality in terms of antibiotic effectiveness. Even in high-income countries, the current generation has unequal access to antibiotic treatment options with respect to the generation who first benefitted from the introduction of effective antibiotics. Similarly, in 30 years, people may have fewer or more options than people presently do. It depends on the success of putting in place effective measures to curb AR. Furthermore, it depends on whether medical research can develop new drugs and treatments (Millar, 2011).

Regarding our responsibility toward society and future generations, ascribing moral responsibility can be said to consist of two acts. First, when stating that someone is responsible, we usually say something about the causal link between the agent and the event or situation for which they are held responsible. Second, we state that they are morally blameworthy for that event or situation. The first statement is seemingly factual, albeit it also includes normative assumptions about what counts as a cause. For example, is AR caused by societal neglect to regulate antibiotics use or do demanding and ignorant individuals cause it? The second is merely normative and can be understood in terms of the moral norms we as a community adhere to (Smiley, 1992). Although these two aspects can be separated in theory, they are often conflated in public debate.

The participants mostly considered the use of antibiotics morally acceptable when it was necessary for one’s care, and morally questionable in all other cases. Presumably, participants were thus implicitly referring to ‘informed’ wrongdoing, i.e. that if one is aware of the problem of AR, then using antibiotics in a non-judicious manner is morally wrong. In discussions concerning moral responsibility, philosophers usually presume that there are two conditions commonly thought to excuse an agent from moral blameworthiness. First, if the agent was not free to act, they are not blameworthy. Second, if the agent lacked relevant knowledge, they are not blameworthy (Fischer and Ravizza, 1998). Concerning the second point, there is also a discussion on culpable ignorance. In some instances, an agent might not have had the relevant knowledge, but they *should* have known it or taken action to get the relevant knowledge, when information can be seen as effortlessly and easily retrievable (Smith, 1983, 2014). As we can see, these discussions are highly relevant to the way in which the participants discussed responsibility. Although at present there is a certain ignorance about the AR problem and the proper use of antibiotics, this ignorance may soon be considered inexcusable (Littmann and Viens, 2015).

The participants believed that as a citizen, you should ‘inform yourself’. If there are causal links between what the individual does and the more significant societal problem of AR, the individual is potentially blameworthy, according to this line of reasoning. Moreover, lack of knowledge may not reduce that responsibility if the information is out there. Considering that it would be challenging and ethically dubious to enforce the promotion of good practices, such as hygiene routines, through privacy infringements, the moral responsibility to act hygienically ultimately rests on the individual. The reason for this is self-protection, but also the duty to spare other people (Parsonage *et al.*, 2017).

The statements expressing the view that individuals should behave in certain ways, educate themselves and develop certain attitudes, can be conceptualized in terms of virtue ethics. Whereas responsibility can refer to obligations and certain actions, it can also refer to a notion of individuals cultivating certain character traits (Nihlén Fahlquist, 2019). Through the lens of this latter notion, one could see responsibility as a virtue, i.e. to develop a notion of responsibility including, for example, a sensitivity to when antibiotics are necessary and to the judicious use of antibiotics. Responsibility as a virtue has been described as a ‘readiness to respond to a plurality of normative demands’ (Williams, 2008), and the complexity of the antibiotics problem could be seen as requiring a certain sensitivity in relation to the plurality of normative demands involved. This can entail actions such as complying with the prescriptions and not interrupting the course of antibiotics as soon as the symptoms disappear, not self-medicate with drugs bought online or using leftovers as soon as symptoms appear. Furthermore, it could include an idea of the right balance between the protection of individuals and being fair to both current and future patients.

The participants envisaged both collective and individual responsibility to ensure antibiotic effectiveness for those who need them the most, now and in the future. They conceived of collective responsibility as being shared by everyone in society, citizens and authorities alike. The participants primarily focused on individual responsibility in a broad sense. As using antibiotics involves a responsibility toward society, and because the demand of society to preserve antibiotic effectiveness should take precedence, individual responsibility was linked to social judgment. In this context, abstaining from using antibiotics was interpreted as a source of relief while their non-judicious use could trigger a feeling of shame. The tension between the individual and the society here involved simultaneously defending the prioritization of society and the treatment needs of vulnerable patients. While maintaining that the behavior of the individual who non-judiciously uses antibiotics is blameworthy, participants were also worried about the stigmatization of socially undesirable behavior. Such a risk of stigmatization is frequently discussed in public health discourse. For example, public health policies aimed at reducing smoking and preventing obesity always run the risk of stigmatizing people. For this reason, a balance has to be struck between the societal needs, e.g. reducing obesity-related diseases and their costs to the community, and the rights and autonomy of, and the effects on, the individual (Guttman, 2000; Nihlén Fahlquist, 2006, 2018; Riley *et al.*, 2017). If people judge others, who take antibiotics, the situation could be similar to the case of, e.g. obese people being judged for overeating. The way in which respondents stated that social judgment is involved indicates that some of the respondents worry about potential emerging stigmatization and shaming of antibiotic users.

The participants lamented the uncertainty of AR risks. Spatial and temporal proximity to the problem would bring an immediate sense of moral responsibility to the participants, while the perceived uncertainty about the risks posed by AR diminished the moral imperative. The moral relevance of visibility was discussed in a recent study in which the authors concluded that where the visible consequences of AR become more evident, the temporal distance between patients is blurred. This should, the authors argue, help to promote a ‘recognition of necessity’ for action to preserve antibiotic efficacy (Krockow and Tarrant, 2019). The fact that participants often used a climate change analogy to describe AR reflects the problem of uncertainty. A problematic aspect is that global warming did not activate our moral intuitions for decades. In the literature, similarities between climate change and AR have been highlighted (Anomaly, 2010; Millar, 2011, 2019). The limit of analogical reasoning being applied to AR is that this reasoning does not capture the complexity and specificity of the AR problem, the risk being an inadequate transfer of solutions from one policy field to another (Littmann and Viens, 2015).

While public health policies can sometimes impose personal sacrifices on individuals, it is often believed that there are limits to the level of demandingness that ‘doing the right thing’ can require of individuals (Giubilini and Savulescu, 2019). Different individuals can interpret the level of demandingness in different ways according, for instance, to their view of individual responsibility. The participants considered it justifiable for health authorities to impose regulations that are more stringent to curb the rise of AR. As noted, they primarily focused on medical prescriptions and the livestock industry and regarded stricter policies regarding these as potentially acceptable changes even if they had significant consequences for citizens. In the following section, we discuss two different theoretical proposals for the preservation of antibiotic effectiveness in light of our findings. The two proposals are Millar’s principle of antibiotic use, and Giubilini and Savulescu’s proposal for an incentive-based policy. These two proposals were chosen because of their relevance in the academic debate and because they present characteristics that appear to fit rather well with the moral views expressed by the participants.

### Two Proposals Discussed

Based on Scanlon’s contractualist approach, Millar suggests the following principle for the fair distribution and constrain of antibiotics:

[A]ntibiotics should be used to prevent some substantial risk of irretrievable harm in patients or their contacts, where a substantial risk is a level of risk that can be reduced by the use of antibiotics, and which exceeds the range of risks of irretrievable harm that we tolerate in our day-to-day lives. (Millar, 2012: 467).

The principle should serve to rule out: (i) entirely inappropriate use of antibiotics and (ii) the use of antibiotics for infections that do not involve a risk of irretrievable harm (see Littmann, 2014). The principle hinges on two underlying assumptions, which appear to match the views of FGD participants well: that antibiotics are a common good and that misusing them goes against the principle of justice. Millar’s principle may at first sight look as a good candidate for accommodating participants’ moral views and, if it accurately captions the public sentiment, a good starting point for an acceptable and useful policy aimed at constraining the use of antibiotics. There are some limitations to this approach, though. The notion of minimal risk, as the one we accept in our day-to-day life, is imprecise. Another potentially problematic aspect is that preserving antibiotics only for the treatment of infections that could cause irretrievable harm would entail leaving many patients untreated. As noted by Littman and Viens (2015), the application of Millar’s principle would expose patients to higher risks of complications, prolonged illness and even higher risk of mortality. On the positive side, using antibiotic treatment only to prevent some substantial risk of irretrievable harm may well prolong antibiotics life and maintain their effectiveness so that future generations could benefit from them. Nonetheless, public health policy inspired by Millar’s principle may be very demanding for patients and may not tally with the public sentiment on the issue. Finally, although FGD participants considered the non-judicious use of antibiotics, together with their irrational prescription, as particularly morally blameworthy, they were also concerned with issues of distributive justice: Who decides who needs antibiotics, according to what criteria and when can antibiotics be considered *really* needed? One could try and use a thought experiment to discuss the matter. The ‘Veil of ignorance’ is a thought experiment developed by Rawls (1971), which can be usefully applied to explore health policy alternatives (Goold, 1996; Korobkin, 1998; Fritz and Cox, 2019). Rawls aimed to describe what free and equal persons, unaware of their position in society, would consider a fair agreement concerning the fundamental principles of justice in society. In such a situation, Rawls argued, people would be able to agree on basic principles of justice (Rawls, 1971). With regards to antibiotic treatment and the maintenance of antibiotic effectiveness, one may believe that behind a veil of ignorance, not knowing whether they would be healthy or affected by an infection or whether they would be patients now or in the future, people would make predictable choices. First, they would possibly argue for the right of future people to have effective antibiotic therapeutic options. Second, they might also agree on policies that would entail less than optimal treatment for present patients. Krockow and Tarrant (2019) resorted to the ‘veil of ignorance’ to explore a scenario in which there would be a choice between different antibiotic treatment approaches, considering current patients with infection symptoms, future patients and non‐infected individuals, independent of one’s personal role. The authors derived, also based on some empirical data (cf. Andersson and Lyttkens, 1999), that ‘most people would generally agree on making appropriate efforts to preserve antibiotic efficacy for future patients through limiting antibiotic use with current patients’ (Krockow and Tarrant, 2019: 758). In substantial agreement with Millar’s principle and, according to Krockow and Tarrant (2019), in line with Rawls’ idea of ‘minimising the worst outcome’, they considered it ethically justifiable to make exceptions only in extreme cases, to prevent patients’ death.

Indeed, Rawls believed that behind the ‘veil of ignorance’ people would minimize the worst outcome and not maximize an aggregate good (Rawls, 1971). While the maintenance of antibiotic effectiveness is a very desirable collective good that people who are ignorant about their position in society and in time probably would try to achieve for everyone, the focal point is what people would intend and try to avert as the worst outcome. Leibovici *et al.* (2012) also explored the issue of antibiotic effectiveness and intergenerational justice by resorting to the ‘veil of ignorance’. They considered as the worst outcome to provide (present) patients with suboptimal treatments and to expose them to higher morbidity and risk of mortality (Leibovici *et al.*, 2012). Therefore, there is doubt whether people behind a veil of ignorance, trying to minimize a worst outcome for themselves, would argue for suboptimal treatments for present patients (i.e. where antibiotic treatment is only acceptable to prevent some substantial risk of irretrievable harm) to benefit future patients—future patients who would be equally exposed to higher risks of complications, longer illness and even higher risk of mortality. Consider how the FGD participants’ primary moral compass did not point at merely maintaining antibiotic effectiveness for as long as possible, so as to maximize the number of patients who could benefit from antibiotics. Instead, participants appeared to think that society should aim to maintain antibiotic effectiveness for as much and as long as possible to benefit all those who need it, including people in the present. The morally salient aspect was the individual need for antibiotic treatment. As highlighted in the previous section, while it is true that participants placed society first, they also thought that the common good should not be sought at the expense of individual patients, implicitly adhering to a layperson’s version of the rescue rule. So, the public would possibly perceive a health program that implemented Millar’s principle as supererogatory. How demanding should a policy aiming at preserving antibiotic effectiveness be?

Giubilini and Savulescu (2019) argue that states should only impose requirements on their citizens that the citizens would have a moral obligation to fulfill irrespective of the state making it mandatory. This would usually result in requirements that would not be too demanding or else in individuals receiving compensation for being coerced to do something supererogatory. Giubilini and Savulescu (2019) regard vaccination as something that puts low demands on individuals and which, therefore, should be pursued even through hard coercion measures. In contrast, foregoing antibiotics can sometimes be very demanding. For this reason, incentives are the preferable option. Incentives can be of an economic nature, as we have argued elsewhere (Ancillotti *et al.*, 2018), but could also consist of increased medical attention to monitor the infection. In addition, the positive influence of social norms suggests social recognition and praise could be a good option. Giubilini and Savulescu’s proposition, i.e. resorting to incentives of various nature to restrain antibiotic use, may be preferable to the previous policy proposal for two reasons. First, it may result in public health programs for the containment of AR that are more in line with the moral intuitions of citizens. Such programs would be compatible with public views on what they owe society and on what justice demands. Second, incentive-based policies still allow people to remain free to decide for or against the incentivized option (Giubilini and Savulescu, 2019).

## Conclusions

The most important finding of this study is that laypeople do understand the ethical dimension of AR and that they are morally sensitive to the problems entailed by the loss of antibiotic effectiveness. This empirical finding suggests that appeals to morality can be included in communications aimed at fostering judicious antibiotic use. Health programs for the containment of AR could be positively received if a moral discourse about antibiotics is developed. AR is a collective action dilemma, i.e. a kind of problem whose solution, or mitigation, is possible only if large enough groups of people share correct behavior. For this reason, it can be influenced by social norms. They hold the potential to contribute to the adoption of individual judicious antibiotic use by promoting virtuous antibiotic use. Highlighting the moral dimension of antibiotic use could also be part of doctor–patient communication, such as in the case of pushy patients. The findings suggest that doctors should not shy away from this idea and might make use of moral nudges.

Concerning the content of this communication, the analysis of the FGDs in this study suggests that lay people believe there is a moral duty to preserve antibiotic effectiveness for present and future patients, based on justice. The non-judicious use of antibiotics is wrong and everyone should assume responsibility for using antibiotics judiciously. While people appear willing to tolerate some personal disadvantage for the benefit of the society, the message that no one is going to be sacrificed for the common good should be clear. Trust in doctors and health authorities, which is a fundamental requisite for promoting the judicious use of antibiotics, could otherwise be undermined.

## Funding

This research received no specific grant from any funding agency in the public, commercial or not-for-profit sectors.

## Conflict of Interest

None declared.

## Footnotes

1 Throughout the article, the distinction between *judicious* and *non-judicious* use of antibiotics has been made. The latter includes overuse, underuse and all types of misuse of antibiotics, such as taking leftovers or non-adherence to the prescription practices.

2 In the exemplar quotes (Q), W and M indicate the participants’ gender and 1–4 their numerical code.
